# Therapy with bone marrow mesenchymal stem cells in bone regeneration in children with osteonecrosis secondary to sickle cell disease

**DOI:** 10.3389/fcell.2024.1410861

**Published:** 2024-05-06

**Authors:** Thiago Rhangel Gomes Teixeira, Gildásio de Cerqueira Daltro, Fernando Luis Sberge, Eduardo Silva Reis Barreto, Antônio Ferreira da Silva

**Affiliations:** ^1^ Postgraduate Program in Biotechnology, Federal University of Bahia (UFBA), Salvador, Bahia, Brazil; ^2^ Department of Experimental Surgery and Surgical Specialties, Federal University of Bahia (UFBA), Salvador, Bahia, Brazil; ^3^ Federal University of Bahia (UFBA), Salvador, Bahia, Brazil; ^4^ Institute of Physics, Federal University of Bahia (UFBA), Salvador, Bahia, Brazil

**Keywords:** hematopoietic stem cells, sickle cell anemia, osteonecrosis, children, bone regeneration

## Abstract

**Introduction:**

This study aimed to describe the evolution of bone regeneration in children with hip osteonecrosis associated with sickle cell disease, treated with bone marrow-derived mesenchymal stem cell implants at the Professor Edgar Santos University Hospital Complex.

**Materials and methods:**

A non-randomized clinical trial was conducted with 48 patients of both sexes, aged between 11 and 18 years, diagnosed with femoral head osteonecrosis secondary to sickle cell disease. Patient selection was based on strict criteria, including confirmed diagnosis of sickle cell anemia and a stage of osteonecrosis compatible with the proposed treatment. Bone regeneration assessment was performed through radiographic examinations and magnetic resonance imaging, following the Ficat & Arlet criteria and the Salter-Thompson classification.

**Results:**

Statistical analysis revealed a significant association between the patients’ age and positive treatment outcomes, suggesting that autologous bone marrow cell implantation is a safe and effective approach in the early stages of osteonecrosis. The majority of patients (87.5%) reported complete pain relief, while 10.42% experienced significant symptom improvement. Only one patient (2.08%) did not observe improvement. The results indicate that cell therapy can regenerate or slow the progression of bone necrosis, reducing the need for more invasive surgical procedures.

**Conclusion:**

The study demonstrates the potential of bone marrow-derived mesenchymal stem cell implantation in treating hip osteonecrosis in children with sickle cell disease, emphasizing the importance of long-term monitoring of bone structure stability.

## 1 Introduction

Sickle cell disease (SCD) is a hereditary disease with autosomal recessive inheritance, where the mutation of hemoglobin can generate complications related to both hemolysis and microvascular occlusion. Conversely, sickle cell anemia (AF) is a type of hemolytic anemia characterized by the presence of red blood cells with altered morphology—sickle-shaped appearance—which are removed from circulation and destroyed at a faster rate (Daltro and Hernigou, 2014). Vascular occlusion caused by the morphological alteration of red blood cells is the main factor in this disease, often leading to ischemia of various organs and tissues (Daltro and Hernigou, 2014). A consequence of this alteration is the loss of elasticity and deformability, leading to increased viscosity in the cytosol and greater adherence of erythrocytes to the endothelium, reducing their fluidity and favoring the formation of thrombi in micro and macrocirculation with consequent vaso-occlusive phenomena (Galiza Neto and Pitombeira, 2003).

The vaso-occlusive complications of AF are much more significant than problems related to anemia, as anemia is generally well tolerated (Ramalho et al., 2003). The respiratory, cardiovascular, musculoskeletal, skin, central nervous system, spleen, kidneys, and liver are most affected by these vaso-occlusive complications (Ramalho et al., 2003). Patients with sickle cell disease present osteoarticular impairment in 80% of cases, in the form of osteomyelitis, synovial joint disease, hemochromatosis, or osteonecrosis (Daltro et al., 2010).

Clinical presentations occur early, with approximately 96% of individuals with SCD developing specific symptoms of the disease by the age of 8 (Baum et al., 1987). Considering that AF primarily affects children and young people, musculoskeletal complications generate a high social and financial burden, as they require permanent medical follow-up and prevent these individuals from having a normal productive life (Cançado and Jesus, 2007). Most of the time, the clinical course of the disease is asymptomatic until the onset of limiting pain, followed by structural bone deformity and loss of joint function in the individual (Daltro and Hernigou, 2014). Hence, early diagnosis is extremely important for safer, more effective, and less invasive treatment before the only treatment alternative is complete joint replacement (Daltro et al., 2008). The use of autologous bone marrow mesenchymal stem cell (BMSC) implantation is an important technique for the future of these patients, as it is minimally invasive and the osteoinductive effect has shown promising results (Daltro et al., 2008).

In Brazil, about 2,500 children are born each year with sickle cell disease (Lobo et al., 2003). In Bahia, the state with the highest prevalence of SCD cases (ranging from 6.9% to 15.4% in individuals of African descent), one in every 650 newborns is a carrier of sickle cell disease, and one in every 27 has sickle cell trait (Silva et al., 2017). In Minas Gerais, the incidence of one case of SCD was reported for every 2,800 births, and in Rio de Janeiro, there was one case for every 1,196 births (Wambier et al., 2007; Daltro et al., 2018a).

It is crucial to highlight initially the central role of the Brazilian Unified Health System (SUS) for the black population compared to the white population throughout Brazil, as public health services are predominantly used by this group. In the Southeast region, for example, the majority of SUS consultations are conducted by black individuals, representing 62.3% of the total, compared to 41.6% of white people. This reliance on public health services among the black population is observed across the country, as evidenced by 2008 data, where 55.2% of consultations were conducted on black and mixed-race individuals, in contrast to 44.1% on white people (Lages et al., 2017).

In SCD, the femoral head is the most common area of destruction (68.5%), followed by the humeral joint as the second most affected area (21.7%), and the knee with approximately 6.5% of cases (Daltro and Hernigou, 2014). Initially, infarctions occur in the subchondral region, where collateral circulation is minimal, and typically involve necrosis of a triangular segment with the subchondral bone plate as its base and the center of the epiphysis as its apex (Powars et al., 2005). The overlying articular cartilage often remains viable in the early stages of the disease, originating from the synovial fluid. In the immediate response, osteoclasts reabsorb the necrotic trabeculae; however, these remain as support for the new living bone deposited in a process known as “Progressive Replacement.” If this process is slow enough, progressive microfractures will occur until collapse of the trabecular bone and ultimately joint necrosis (Powars et al., 2005; Gruson and Kwon, 2009). The high hematocrit concentration among patients with SC genotype favors the onset of osteonecrosis due to increased intraosseous vascular stasis (Flouzat-Lachaniete et al., 2009).

It is also presumed that the initial event is the obstruction of medullary sinuses by sickle cells, leading to necrosis of the bone marrow and the cells forming the bone tissue (Manwani and Frenette, 2013). This necrosis would trigger a bone repair process that, despite improving the injury, especially in young individuals, would also lead to increased intramedullary pressure, resulting in bone resorption and structural collapse (Manwani and Frenette, 2013). This diagnosis is initially based on clinical criteria. Manifestations include persistent pain, decreased range of joint motion (beginning with limitation in hip internal rotation), and, in more advanced stages, the occurrence of joint ankylosis. Commonly, the identification of these symptoms signals that the pathology has already reached an advanced stage of development. Therefore, there is a critical need for early diagnostic interventions in patients affected by sickle cell anemia, aiming to detect osteonecrosis before the manifestation of indicators of progression to advanced stages of the disease.

The quality of life of children diagnosed with avascular necrosis of the femoral head reveals a significant inferiority when compared to those without this condition. This disparity was evident in all domains analyzed by a specific questionnaire, which encompasses psychosocial areas, physical capacity, emotional aspects, social interactions, and academic performance. The most pronounced impact on HRQoL was observed in the domain of physical capacity, while the least impairment was observed in the emotional domain (Matos et al., 2014).

Bone marrow aspirate from the iliac crest of patients with SCD contains mesenchymal progenitor cells with osteogenic and chondrogenic potential, as well as endothelial progenitor cells capable of contributing to vasculogenesis and angiogenesis, leading to vascular repair (Daltro et al., 2008). Among the various etiologies contributing to osteonecrosis, patients with SCD have the best prognoses for autologous stem cell-based therapy, with a failure rate below 5%, resorting to shoulder arthroplasty after 8 years (Daltro et al., 2008; Daltro and Hernigou, 2014). This study aims to investigate the use of autologous BMSC implantation as a therapeutic approach for osteonecrosis in patients with SCD.

## 2 Materials and methods

### 2.1 Study design

This is a Non-Randomized Clinical Trial conducted in patients from various regions of Brazil, aged between 11 and 18 years, with femoral head osteonecrosis, who were admitted for treatment and surgical intervention at the Professor Edgard Santos University Hospital Complex (Com-HUPES).

### 2.2 Population selection

Participants were recruited through referrals from other healthcare institutions via state regulation or direct referral from Hemotherapy Centers. A total of 48 patients with a diagnosis of osteonecrosis secondary to Sickle Cell (SC) or Sickle (SS) Anemia, of both sexes, seen at the Magalhães Neto outpatient clinic, annexed to the Professor Edgard Santos Hospital Complex, between the years 2008 and 2023, were included.

The diagnosis of ONFH was determined by radiographic imaging examination in anteroposterior and lateral planes. When patients with SCD had moderate to severe pain localized in the hip or groin region, and physical examination showed limited movement of the affected hip, while radiography showed no abnormalities, the diagnosis of ONFH was made by magnetic resonance imaging (MRI) at the beginning of treatment. Radiological progression was determined according to the development of the Ficat stage and the Salter-Thompson classification, with the aim of determining the following characteristics: presence of femoral head collapse; presence of a sclerotic band in the femoral head; increasing signal; double signal in T2 on magnetic resonance imaging; cyst or sclerosis in the femoral head; hip pain on movement; focus of low intensity in T1.

### 2.3 Inclusion criteria

To be included in the study, patients had to meet the following criteria.

1. Confirmed diagnosis of Sickle Cell Anemia, whether homozygous or heterozygous, by hemoglobin electrophoresis.
2. Age between 11 and 18 years at the time of inclusion.
3. Presentation of exams allowing the classification of osteonecrosis at a stage still amenable to intervention with the proposed treatment.

### 2.4 Exclusion criteria

Patients who met one or more of the following conditions were excluded from the study.

1. Diagnosis of osteonecrosis associated with the use of corticosteroids or other causes besides Sickle Cell Anemia.
2. Lack of confirmation of the diagnosis of Sickle Cell Anemia through hemoglobin electrophoresis.
3. Age younger than 11 years or older than 18 years at the time of inclusion.
4. Presence of osteonecrosis at an advanced stage, indicating the need for prosthesis implantation.
5. Patients with surgical site infection or systemic infection.
6. Patients with fracture resulting from trauma.

### 2.5 Data collection instruments

For data collection, a clinical information extraction form of the patients was used. This form contained the following variables.

1. Year of diagnosis of Sickle Cell Anemia.
2. Age at the onset of symptoms.
3. Time elapsed until the diagnosis of osteonecrosis.
4. Results of imaging tests performed before and after treatment (immediately after surgical intervention, at 6 months, and 1 year).
5. Dominance of osteonecrosis.
6. Exact location of the bone lesion.
7. Specific classification of the severity of osteonecrosis in the affected follow-up.

### 2.6 Methods of obtaining and processing the cellular aspirate

**FIGURE 1:**
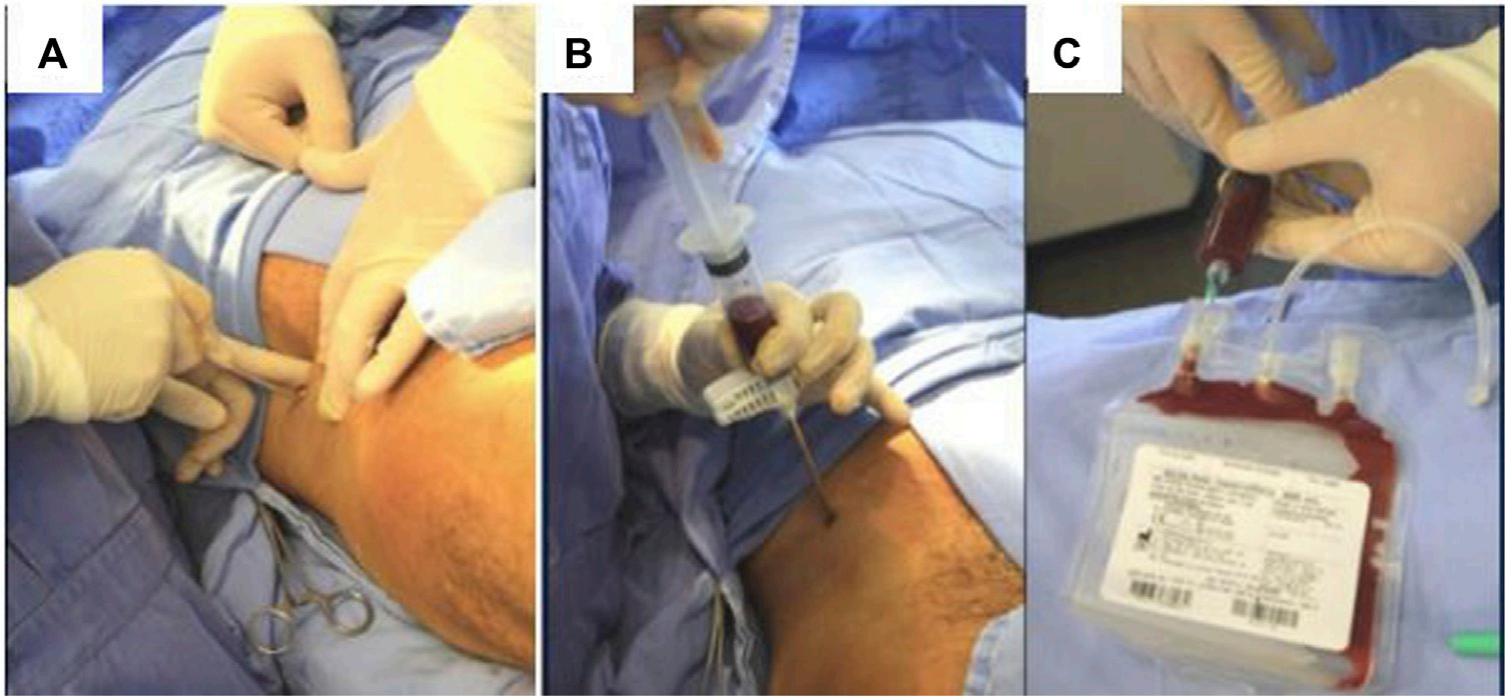
Methods for obtaining and processing cell aspirate/**(A)** Small incision over the skin; **(B)** Bone marrow aspiration at the iliac crest. **(C)** Collection plastic bag containing culture medium and anticoagulant solution.

After all preparation and the use of aseptic and antiseptic techniques, surgeries performed at the COM-HUPES began, under anesthesia in the operating room, with a small incision on the skin, and subsequent introduction of a Jamshidi-type needle, with puncture and aspiration of bone marrow, at the patient’s ipsilateral iliac crest (Figure 1A).

**FIGURE 2:**
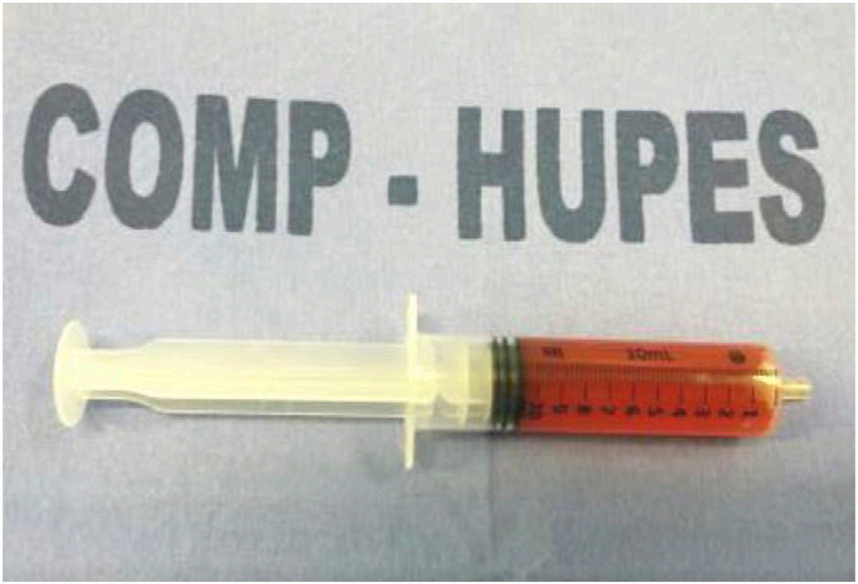
Syringe containing autologous mononuclear cell concentrate.

A total of 100 mL of bone marrow aspirate (BMA) was obtained by puncture and aspiration of the anterior iliac crest of patients under general anesthesia. The marrow was aspirated in small fractions at a time [20 mL per heparinized plastic syringe (Figure 1B)] to reduce dilution by peripheral blood and to maximize the number of progenitors at the graft site, then temporarily stored in a sterile collection bag (Figure 1C). The bone marrow mononuclear cells (BMMCs) were concentrated using the SEPAX cell separator (Biosafe, Eysins, Switzerland) according to the manufacturer’s recommendations. After centrifugation, a final volume (∼40 mL) of concentrated bone marrow mononuclear cells in suspension with 5% human serum albumin was collected and placed in syringes for reinjection during the same operation (Figure 2). A small fraction of the BMA and BMMCs was separated for flow cytometry, cell viability, and microbiological assays.

### 2.7 Surgical procedure for cell suspension injection

**FIGURE 3:**
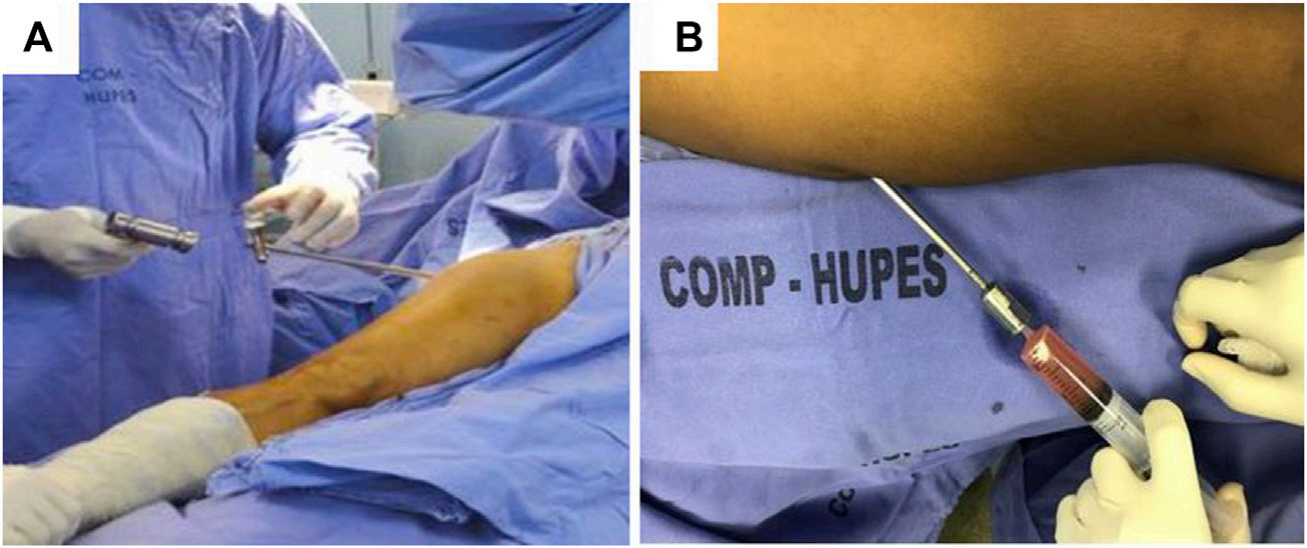
Methods of obtaining and processing cell aspirate 2/**(A)** Patient positioning and material collection **(B)** Injection of autologous mononuclear cell concentrate into the femoral head.

Immediately following the completion of the bone marrow puncture and aspiration procedure—while still under anesthesia - the patient was positioned in a dorsal decubitus at a 45° semi-flexion with the image intensifier contralateral to the operated limb (Figure 3A).

**FIGURE 4:**
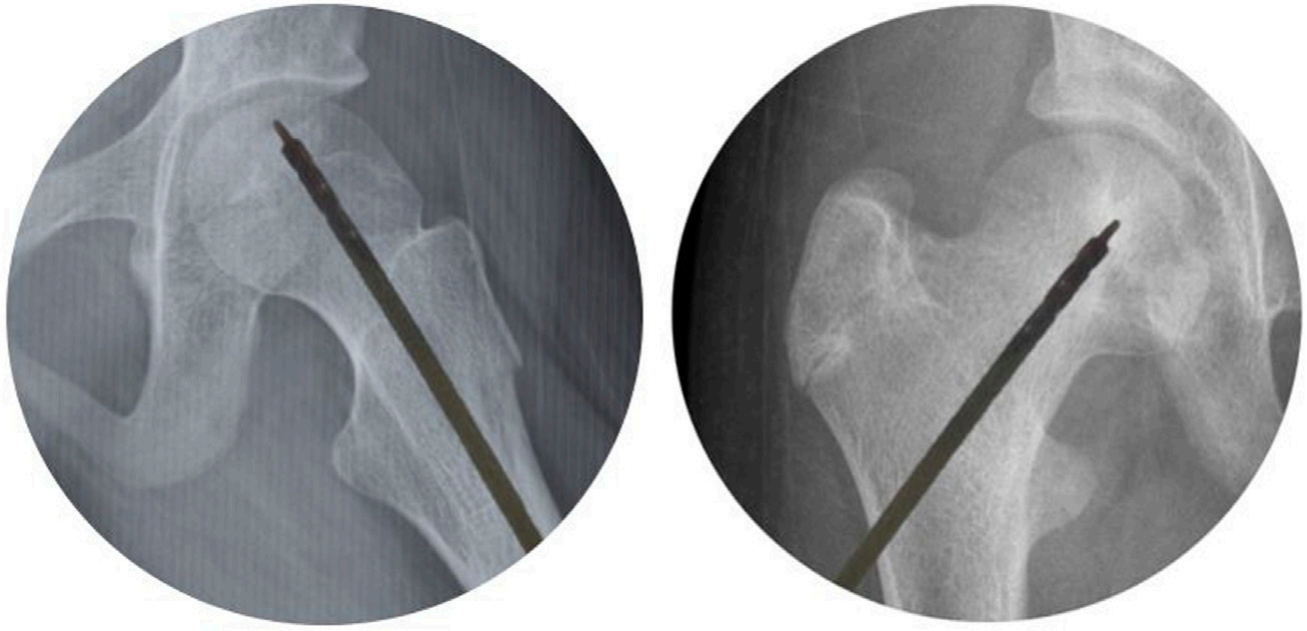
Intraoperative use of fluoroscopy.

After appropriately positioning the patient, the BMMCs were percutaneously injected using a 1 mm diameter trocar (Mazabraud trocar, Collin, France) into the center of the osteonecrotic area. Each patient received the BMMC infusion through a single injectable puncture (Figure 3B). The procedure was pre-planned using nuclear magnetic resonance images, and the needle’s position in the femoral head area was monitored by fluoroscopy (Figure 4).

### 2.8 BMMC clones assays

Fibroblast unit colony-forming assays (CFU-F) were conducted to determine the number of mesenchymal progenitors in CMMO samples, as previously described (Ahrens et al., 2004). Briefly, single-cell suspensions of BMMCs were cultured in 1.0 × 106 cells/25 cm2 flasks with low-glucose Dulbecco’s Modified Eagle Medium (DMEM) (Sigma, St. Louis, MO, United States), supplemented with 20% fetal bovine serum (Cultilab, Campinas, Brazil), 100 U/mL penicillin, and 100 μg/mL streptomycin (Invitrogen, Grand Island, NY, United States), and incubated in 5% CO2 at 37°C. After 10 days, non-adherent cells were removed by washing with phosphate-buffered saline, and the remaining adherent cells were fixed in 4% buffered formalin for 10 min. To detect CFU-F, cells were stained with Crystal Violet solution. Cultures were classified into clusters (20–50 cells/cluster) and colonies (>50 cells) under ×10 magnification microscopy. To determine the potential for colony-forming osteoblasts (CFU-O) of CMMOs, cells were cultured in basal medium containing 10 nM dexamethasone (Sigma), 5 mM sodium β-glycerophosphate (Sigma), and 50 μg/mL ascorbic acid-2-phosphate (Sigma), and incubated as described (Baksh et al., 2003). After 21–24 days in culture, CFU-Os were fixed in 4% buffered formalin for 10 min and stained with Alizarin Red S for mineralization.

### 2.9 Immunophenotyping

BMMCs and BMA were analyzed for the expression of cell surface antigens using direct three-color analysis with fluorescein isothiocyanate (FITC)-conjugated, phycoerythrin (PE)-conjugated, and peridinin chlorophyll protein (PerCP)-conjugated monoclonal antibody complexes as previously reported (Fadini et al., 2007; Nevskaya et al., 2008). The following antibodies were used for analysis: PE-conjugated immunoglobulin G1 control (eBioscience, San Diego, CA, United States), PerCP-conjugated immunoglobulin G1 control (Becton Dickinson, San Jose, CA, United States), FITC-conjugated immunoglobulin G1 control (BD Bioscience, Franklin Lakes, NJ, United States), anti-CD34-FITC (BD Bioscience), anti-CD34-PerCP (Becton Dickinson), anti-KDR-PE (R & D Systems, Minneapolis, MN, United States), anti-CD45-PerCP (BD Bioscience), and anti-CD133-PE (Miltenyi Biotec, Bergisch Gladbach, Germany). The quantification of total mononuclear cells in CMMOs and BMA was determined by Turk’s solution. For FACS analysis, 5 × 105 events were acquired and analyzed with a FACSCalibur analyzer (Becton Dickinson). Data were processed using the Macintosh CELLQuest software program (Becton Dickinson).

### 2.10 Viability of BMMCs and quality control

The viability of infused cells, determined by trypan blue exclusion, was over 95%. The study of potential microbiological contaminants, monitored by blood culture and traditional microbiological tests, showed negative results.

### 2.11 Isolation and culture of primary human MSCs

BMMCs were plated at a density of 1.6 × 105 mononuclear cells/cm2 in low-glucose DMEM as described above. After 4-5 days of culture, non-adherent cells were discarded, and the medium was refreshed twice a week. Upon reaching 90% confluence, cells were sub-cultured using 0.05% trypsin-0.02% EDTA at a ratio of 1:3. MSCs were used up to passage six for all studies.

### 2.12 Immunophenotypic characterization of MSCs

The MSC population expanded in the third passage in cell culture was characterized by morphology (spindle-shaped cells) and by immunophenotypic analysis for the expression of the following membrane markers: mouse anti-human CD14-PE (clone 61D3), CD29-FITC (clone TS2/16), CD90-FITC (clone eBIO5E10), and CD105-PE (clone SN6) (from eBioscience), CD31-FITC (clone WM59), CD34-FITC (clone 8G12), CD45-PerCP-Cy5.5 (clone MOPC-21), and CD146-PE (clone P1H12) (from BD Biosciences, San Jose, CA, United States). The detection of osteocalcin in MSC cultures by flow cytometry was performed with mouse anti-human PE-conjugated monoclonal antibody (R&D system) or control isotype antibody according to the manufacturer’s protocol.

### 2.13 Osteogenic differentiation of MSCs and matrix quantification

For osteogenic differentiation, 5.0 × 104 MSCs were plated in a 24-well plate in triplicate. Osteogenic differentiation was induced by culturing confluent MSCs in basic DMEM medium (4 mmol/L L-glutamine/penicillin/streptomycin, 10% fetal bovine serum) supplemented with 100 nmol/L dexamethasone (Sigma), 5 mM β-glycerophosphate (Sigma), and 50 μg/mL ascorbic acid 2-phosphate (Sigma) for 21 days, with media change every 3-4 days. After differentiation, cells were fixed for 10 min with ice-cold 70% ethanol and subsequently stained with Alizarin Red (Sigma; 1% Alizarin Red in distilled water, pH 4.2) to indicate calcium deposition and mineralized nodules within the extracellular matrix (Lee et al., 2006). Morphological assessment of the cultures was performed using an inverted microscope with LED DM IL (Leica, Wetzlar, Germany) coupled to a digital camera for image capture. Quantitative analysis of the amount of Alizarin staining was performed with Fiji (NIH, Bethesda, Maryland, United States). Five fields were selected, and the area of newly formed mineralized tissue in each field was calculated and shown as a percentage of the total tissue area.

### 2.14 Adipogenic differentiation of MSCs

MSCs were seeded in 24-well tissue culture plates at a density of 5.0 × 10^4 cells/well. Adipogenic differentiation was induced in confluent MSCs incubated with basic DMEM medium supplemented with 10 μg/mL insulin, 500 μmol/L three-isobutyl-1-methylxanthine, 100 μmol/L indomethacin, and 1 μmol/L dexamethasone. After 3 weeks of adipogenic stimulation, cells were fixed with 4% paraformaldehyde for 30 min, washed with sterile water, and incubated with 0.5% Oil Red O in isopropanol for 20 min at room temperature. After staining, cells were washed with 60% isopropanol and rinsed with sterile water before being observed under a microscope for image acquisition (Lee et al., 2006).

### 2.15 Chondrogenic differentiation

Chondrogenic differentiation was performed using micromass cell cultures under chondrocyte differentiation medium (Invitrogen) for 30 days. One million MSCs were sedimented at 300 g, and chondrocyte differentiation medium was added without disturbing the sediment. The media were changed every 48 h. After differentiation, cells were fixed with 4% paraformaldehyde and embedded in paraffin. Deparaffinized 5 μm sections were stained for proteoglycans using 3% Alcian blue. After staining, sections were washed with distilled water, air-dried at room temperature, immersed in xylene, and mounted for microscopy. Images were captured using a Nikon Ti Eclipse microscope (Tokyo, Japan).

### 2.16 Quantitative RT-PCR

To determine the relative expression levels of osteogenic markers, 3.0 × 105 MSCs were seeded in 25 cm2 flasks and maintained under control conditions or osteogenic differentiation medium. After 7 days, cells were washed with chilled phosphate-buffered saline, and total mRNA was isolated using RNeasy^®^ mini kit silica columns (Qiagen, Hilden, Germany), following the manufacturer’s protocol. The mRNA concentration was determined by absorbance at 260 nm, and the purity of the preparations was evaluated by the A260 nm/A280 nm ratio using a Nanodrop ND-1000 spectrophotometer (NanoDrop Technologies, Rockland, DE, United States). cDNA was synthesized from 1 μg of total RNA using Superscript II reverse transcriptase (Invitrogen), followed by RNA digestion with RNase H (Invitrogen). The purity of cDNA was confirmed by PCR for glyceraldehyde-3-phosphate dehydrogenase (GAPDH).

Quantitative RT-PCR was performed using the ABI Prism 7,000 sequence detection system (Applied Biosystems, Foster City, CA, United States). All samples were analyzed in triplicate in 96-well plates. The quantitative RT-PCR reaction was carried out using 6 µL of SYBR^®^ Green dye (Applied Biosystems), 3 μL of 30-fold diluted cDNA, and 3 μL of a mixture containing forward and reverse primers (see Additional File 2) and incubated under the following conditions: 2 min at 50°C, 10 min at 95°C, followed by 40 cycles of 15 s at 95°C and 60°C for 1 min. GAPDH, HPRT, and RN18S1 were used as reference genes. Relative expression levels of each gene were analyzed using the 7,300 System SDS software (Applied Biosystems) with the 2^−ΔΔCT^ method (Livak and Schmittgen, 2001). Data are presented as mean 2^−ΔΔCT^ ±standard error. The experiment was repeated using MSCs from three different donors.

### 2.17 Cytokine assays

MSCs (3.0 × 10^4^/cm^2^) were seeded in 24-well culture plates. After 48 h, supernatants were collected, and the levels of transforming growth factor (TGF)-β (Invitrogen), interleukin (IL)-8 (Invitrogen), vascular endothelial growth factor (VEGF; R&D Systems), and stromal cell-derived factor (SDF)-1 alpha (R&D Systems Inc.) were measured by enzyme-linked immunosorbent assay kits according to the manufacturer’s instructions.

### 2.18 Statistical analysis

The collected data were stored in a database created in Microsoft Excel, Microsoft 365 version. Descriptive analysis, including absolute and relative frequency, arithmetic mean, and standard deviation, was conducted to identify the general and specific characteristics of the study sample. For continuous variables such as age and imaging test results, measures of central tendency and dispersion were calculated. Additionally, categorical variables such as gender and treatment outcomes were summarized using frequency distributions.

To assess the association between categorical variables, chi-square tests were performed. Specifically, chi-square tests were used to evaluate the relationship between treatment outcomes and gender, as well as between treatment outcomes and age groups. The significance level was set at 5%.

For continuous variables, such as age, where normality assumptions were met, parametric tests like t-tests or analysis of variance (ANOVA) were employed to compare means between groups, such as treatment outcomes or different age ranges. Non-parametric tests like the Mann-Whitney U test or Kruskal-Wallis test were used when normality assumptions were violated.

Imaging tests performed before, during, and after surgeries were analyzed and compared using appropriate statistical tests, such as paired t-tests or Wilcoxon signed-rank tests for within-group comparisons, and independent t-tests or Mann-Whitney U tests for between-group comparisons, depending on the distribution of the data.

The degree of bone degeneration or regeneration observed in imaging tests was qualitatively assessed and recorded accordingly, with any notable findings documented for further analysis and interpretation. Additionally, regression analysis, such as logistic regression, may be considered to explore the relationship between predictor variables and treatment outcomes while adjusting for potential confounding factors.

### 2.19 Ethical considerations

This study is part of the project entitled: “Analysis of the clinical-epidemiological profile of patients followed up in the orthopedics and traumatology service of the Professor Edgard Santos Hospital Complex.” It obtained approval from the Research Ethics Committee of the Professor Edgard Santos University Hospital, under the protocol number: 3,460,241 and Certificate of Presentation for Ethical Appreciation—CAAE: 13790619.6.0000.0049. The work was conducted in accordance with resolutions 196/96, 340/04, 347/2005, and 466/2012 of the National Commission for Ethics in Research (CONEP) and their complements.

## 3 Results

**FIGURE 5:**
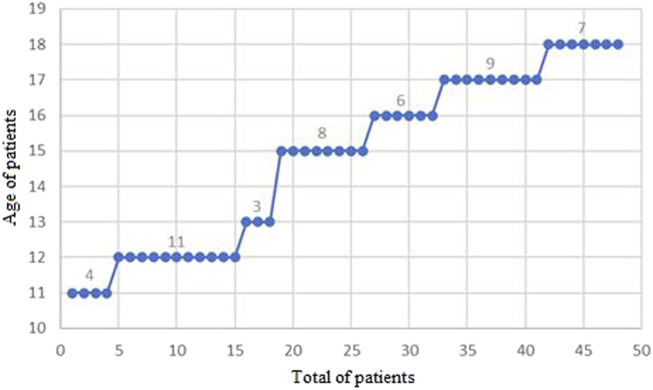
Distribution of patients treated at the Magalhães Neto Outpatient Clinic between 2008 and 2023, categorized by age.

A total of 48 patients, of both genders, were evaluated at the Magalhães Neto Outpatient Clinic during the period from 2008 to 2023. Table 1 provides a descriptive summary of the data regarding the age of the patients under analysis. It is noteworthy that the minimum recorded age was 11 years, while the maximum reached 18 years. On average, the age of the patients was around 15 years, with a mode of 12 years of age (11 cases), and the median in this studied population was 15 years (Figure 5). The analysis of the data distribution revealed negative skewness and kurtosis.

**TABLE 1:** Descriptive summary of the age of patients attended at the Magalhães Neto Outpatient Clinic between the years 2008 and 2023.

Maximum	Minimum	1st quartile	3rd quartile	Mean	Standard deviation	Skewness	Kurtosis
18.00	11.00	12.00	17.00	14.79	2.43	*−*0.19	*−*1.51

**FIGURE 6:**
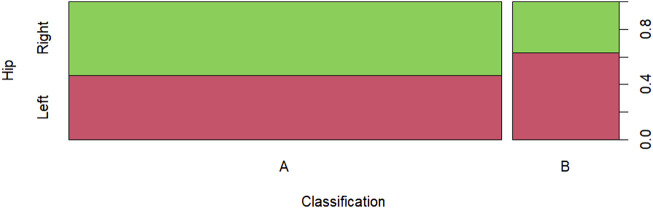
Treatment outcome by gender.

In Figure 6, the graph of treatment outcomes with respect to gender is presented, showing higher percentages for men when the outcomes were stabilization and collapse and a higher percentage for women when the outcome was improvement.

Table 2 presents the quantitative and percentage values (in parentheses) of treatment outcomes by gender. Statistically, we can conclude, with 5% significance, that there is no evidence to suggest that treatment outcome depends on gender, based on the chi-square statistic, with a *p*-value of 0.7694 > 0.05.

**TABLE 2:** Gender vs. treatment outcome.

Gender	Treatment result		
	Stabilization	Improves	Collapse
Male	10_(71*,*44%)_	2_(14*,*28%)_	2_(14*,*28%)_
Female	22_(64*,*70%)_	8_(23*,*53%)_	4_(11*,*76%)_

**FIGURE 7:**
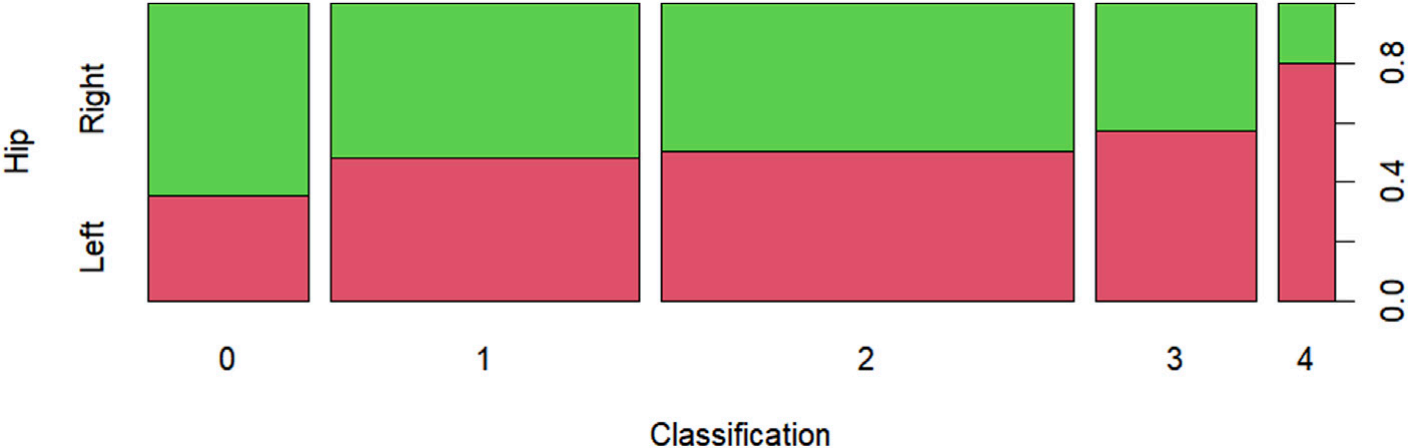
Salter-Thompson classification by hip.

Figure 7 displays the Salter-Thompson classification by hip, showing a greater disproportion in classification B compared to others.

Table 3 presents the quantitative and percentage values (in parentheses) of the Salter-Thompson classification by hip. Statistically, we can conclude, with 5% significance, that there is no evidence to suggest an association between the Salter-Thompson classification and the hip, based on the chi-square statistic, with a *p*-value of 0.3055 > 0.05.

**TABLE 3:** Salter-Thompson classification by hip.

Hip	Classification	
	A	B
Right	41_(53*,*25%)_	7_(36*,*84%)_
Left	36_(46*,*75%)_	12_(63*,*16%)_

**FIGURE 8:**
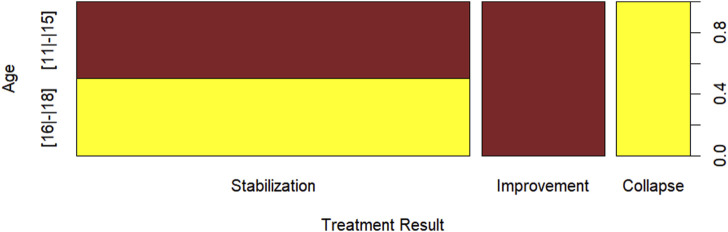
Ficat & Arlet classification by hip.

In Figure 8, the Ficat & Arlet classification by hip is presented, showing a proportional balance in classification two and greater proportional imbalance in classification 4. The graph depicts the proportion of osteonecrosis stage classification per hip based on the Ficat & Arlet classification of 48 patients, of both genders, treated at the Magalhães Neto Clinic between 2008 and 2023. We can observe a higher proportion in stage 0 in the right hip, a proportional balance in stages 1 and 2, and a higher proportion in stages 3 and 4 in the left hip.

Table 4 presents the quantitative and percentage values (in parentheses) of the Ficat & Arlet classification by hip. Statistically, we can conclude, with 5% significance, that there is no evidence to suggest an association between the Salter-Thompson classification and the hip, based on the chi-square statistic, with a *p*-value of 0.5144 > 0.05.

**TABLE 4:** Ficat & Arlet classification by hip.

Hip	Classification				
	0	1	2	3	4
Right	9_(64*,*28%)_	14_(51*,*85%)_	18_(50*,*00%)_	6_(42*,*86%)_	1_(20*,*00%)_
Left	5_(35*,*71%)_	13_(48*,*15%)_	18_(50*,*00%)_	8_(57*,*14%)_	4_(80*,*00%)_

**FIGURE 9:**
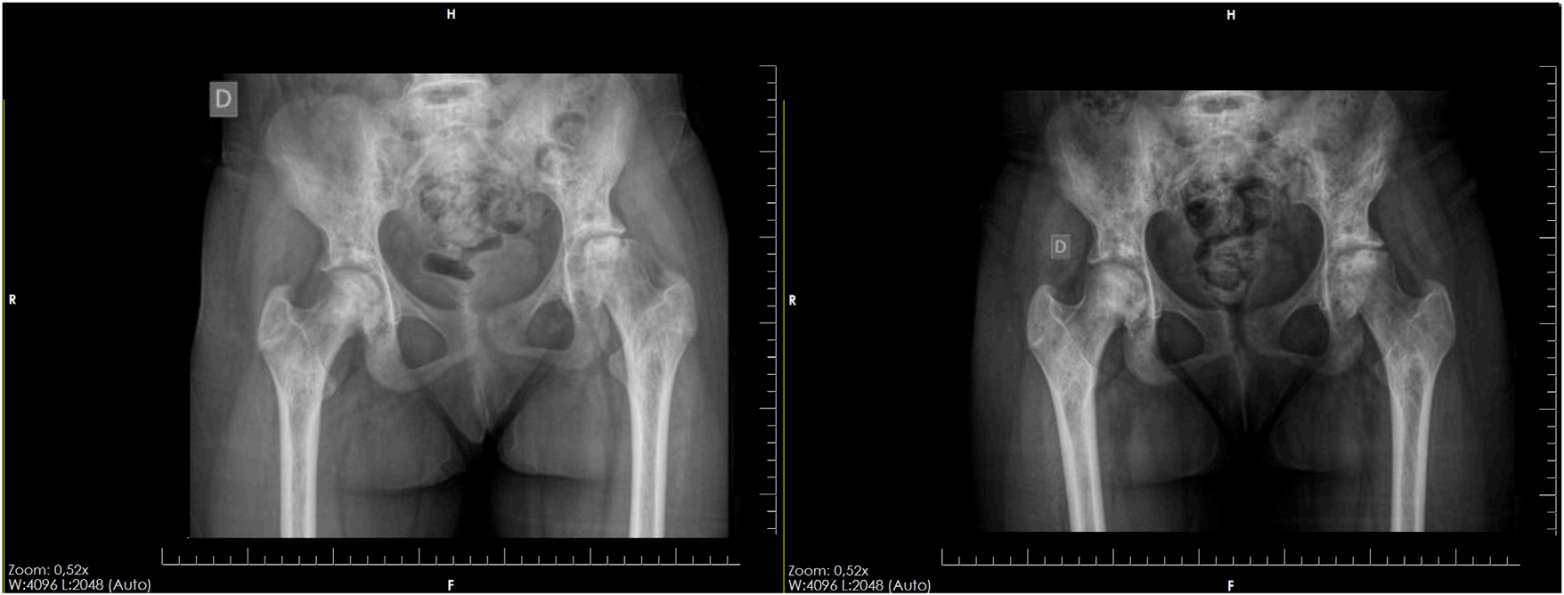
Age x treatment outcome.

Figure 9 presents the treatment outcome by age, where it can be observed that there were no patients aged between 11 and 15 years with a treatment outcome of collapse, no patients aged between 16 and 18 with an outcome of improvement, and a balance in the number of patients with a stabilization treatment outcome between patients aged (11–15) and (16–18).

Table 5 presents the quantitative and percentage values (in parentheses) of the treatment outcome by age. Statistically, we can conclude, with 1% significance, that there is evidence to suggest an association between age and treatment outcome, based on the chi-square statistic, with a *p*-value of 0.0003 < 0.01.

**TABLE 5:** Age x treatment outcome.

Age	Treatment outcome		
	Stabilized	Improves	Collapse
[11|-|15]	16_(61*,*54%)_	10_(38*,*46%)_	0_(00*,*00%)_
[16|-|18]	16_(72*,*73%)_	0_(00*,*00%)_	6_(27*,*27%)_

The analysis of association or dependence in contingency tables, involving categorical variables, is one of the first steps in proposing regression models where the response variable exhibits such characteristics. Considering the result presented in Table 5, indicating an association between age and treatment outcome, we consider the possibility of modeling treatment outcome as a function of patient age. Therefore, Table 6 presents the estimates, standard error, and *p*-value of the multinomial logistic regression model. Both the constant term and the parameter associated with the age variable are significant at the 5% significance level, as shown in Table 6. In terms of interpretability, the estimated coefficient associated with the age variable is of greater interest to us, considering the odds ratio. Consequently, in Table 6, we note that the return includes treatment outcomes two (improvement) and 3 (collapse), meaning our reference is treatment outcome 1 (stabilization). Therefore, for every 1 year increase in the age variable, the odds of the treatment outcome being two (improvement) instead of 1 (stabilization) is exp (−0.860) ≈ 0.4232, while the odds of the treatment outcome being 3 (collapse) instead of 1 (stabilization) is exp (−0.860) ≈ 4.003.

**TABLE 6:** Coefficients, Standard Error, and *p*-value of the Multinomial Logistic Regression Model.

Treatment outcome	Coefficients		Standard error		*p*-value	
	Constant	Age	Constant	Age	Constant	Age
2	10.314	*−*0.860	3.898	0.309	0.008	0.005
3	*−*24.901	1.387	11.435	0.659	0.029	0.035

Regarding the clinical improvement of symptoms post-surgical treatment with stem cells, out of the 48 patients evaluated in this study, 42 reported complete relief of pain (87.5%), five reported significant improvement in pain (10.42%), and only one patient reported no improvement in the presented condition (2.08%). This alone demonstrates the individual and social importance of this treatment approach.

Complications such as fracture, dislocation, nerve or muscle injuries, clinically significant thromboembolism, or hematoma were not observed during or after treatment. No patient required further surgery.

## 4 Discussion

The results suggest that autologous bone marrow cell implantation appears to be a safe and effective approach in treating early stages of osteonecrosis. Consistent with the work presented above, of the patients evaluated in this study, nearly 90% reported complete pain relief, with only 2% of patients showing no improvement in their condition.

The study by Daltro et al. (2015) describes a 5-year clinical trial involving 89 patients with femoral head osteonecrosis secondary to sickle cell anemia. All patients were in pre-collapse stages of femoral head according to the Ficat classification (stages 0, I, or II). The results revealed that none of the patients who were in stages 0/I previously had clinically progressed to femoral head collapse by the end of this period. Only three out of the 89 treated hips did not report clinical improvement after treatment; however, it is important to note that there was also no disease progression to the collapse stage. Therefore, we can analyse that in the pediatric group, postoperative control is challenging, as in some cases, children/adolescents may not understand the importance of maintaining medical measures with the operated limb in zero load during the first month post-surgery, and even then, gradually progress to loading for a gradual return to activities and ambulation in the following months.

The most important indicator for evaluating the success of cell therapy in femoral head osteonecrosis is the ability to regenerate or delay the progression of bone necrosis, reducing or postponing the need for more invasive surgical procedures, such as total hip arthroplasty. Although the results of studies are encouraging, improvement in bone structure is less evident than the clinical improvement of patients; however, in the pediatric population, better results with radiological evidence of femoral bone tissue regeneration are expected approximately 12 months after therapy. The stability of bone structure should be monitored over several years.

As decompressive therapy alone does not prevent the frequent progression of subchondral femoral head fractures in patients with sickle cell disease (Neumayr et al., 2006), our data suggest that concentrated bone marrow mononuclear cell (BMMC) implantation with a minimally invasive technique may be effective in preventing the natural progression to collapse of ONFH and help avoid joint replacement in patients with sickle cell disease. Al Omran (2013) compared the efficacy of decompressive methods for treating pre-collapse ONFH in a consecutive series of sickle cell disease patients. He observed significant pain improvement at the end of 2 years for stage I Ficat patients, but the clinical failure rate, measured as progression to the final stage of ONFH requiring hip arthroplasty, still reached 20%–50% in stage IIA/IIB patients. Thus, central decompression alone may delay the natural progression to collapse in sickle cell disease patients but still has a high failure rate without bone marrow grafting.

Our findings are consistent with those reported by Hernigou et al. (2008) who found greater clinical improvement, pain reduction, delay in disease progression, and absence of subchondral femoral head fractures in sickle cell patients treated with minimally invasive decompression and autologous bone marrow concentrate. The efficacy of BMMC implantation in ONFH was reported for all patients postoperatively over a period of up to 17 years with the longest follow-up time and an average period of 10 years for 87% of hips (Hernigou et al., 2008).

Furthermore, the development of multifunctional hydrogel systems capable of rapid gelation and *in situ* spraying offers promising opportunities to augment the therapeutic capabilities of mesenchymal stem cells (MSCs) in tissue repair and regeneration (Li et al., 2024). Integrating insights from this innovative hydrogel technology into future MSC-based therapies may pave the way for addressing the pressing clinical demands associated with intricate tissue injuries.

**FIGURE 10:**
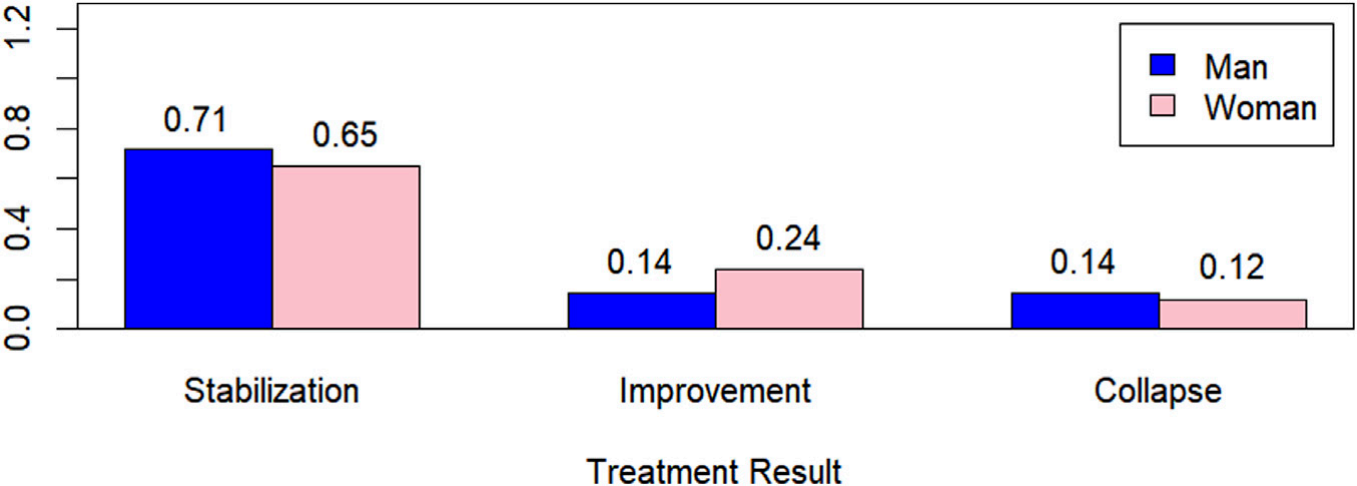
Pelvic radiograph of a 12-year-old child, showing significant improvement of advanced osteonecrosis in the left hip after 1 year of progression.

In our study, further corroborating findings from important literature sources (Daltro et al., 2008; Hernigou et al., 2008; Daltro et al., 2015; Daltro et al., 2018b), nearly 70% of cases stabilized, meaning there was no disease progression, which is very important for these patients, especially considering their young age. Thus, we were able to postpone the need for prosthesis for approximately 10 years (Hernigou and Beaujean, 2002). Among the studied patients, 10 cases (20.83%) showed disease regression, with evident improvement in bone stock and regression of osteonecrosis on imaging examination, highlighting the success of this treatment in children who are still in the early stage of osteonecrosis, as evidenced by the regeneration in a 12-year-old child in 1 year of follow-up (Figure 10).

Significant research by Daltro et al. (2008) illustrates how such monitoring can unveil more promising outcomes. Over a span of 4 years, notable regeneration in the femoral head is evident, transitioning from FICAT IV to FICAT III classification. Regarding the unsatisfactory outcomes presented, six patients experienced femoral head collapse after the end of the study; however, among these patients, five already had such collapse before treatment. It is already known that in advanced cases, stem cell surgery is effective in partially or totally relieving pain, which is of great benefit to the patient (Daltro et al., 2015). Only one case showed progression, with worsening of the condition, which was a patient aged 18, who progressed from Ficat III to IV during treatment, which could be due to numerous factors, such as maintaining weight-bearing on the affected limb contrary to the given instructions, or the disease progression itself not responding to treatment.

The results presented in the Ficat & Arlet and Salter-Thompson classifications reveal a significant proportion of operable cases, with 80.21% of patients indicated in both classifications. These high operability rates have profound implications in clinical practice and patient management for patients with SCD-related diseases. First, the validity and reliability of these classifications are crucial. The accuracy of the classifications in identifying operable cases deserves careful analysis, despite both presenting the same proportion and outcome. It emphasizes the importance of evaluating sensitivity and specificity, as well as predictive values and the patient’s clinical picture, especially in pediatric cases.

Operated on patients with Ficat & Arlet IV and Salter-Thompson B classifications, aiming to delay prosthetic intervention in pediatric cases. While not expecting bone regeneration, surgery addressed pain and prosthetic necessity. The predominance of Black and mixed-race participants underscores the treatment’s importance in high-risk populations with prevalent sickle cell anemia. However, the interplay between the Black population and their physical occupations emphasizes the multifaceted nature of health determinants, extending beyond biology to include social factors. These findings highlight the necessity for tailored healthcare interventions that address both the biological and socio-economic aspects of the disease, particularly in vulnerable populations.

The results indicate that, in many cases, stem cell therapy was able to stabilize the condition or even reverse osteonecrosis, delaying the need for more invasive surgical procedures, such as total hip arthroplasty. This is particularly significant for young patients, as the treatment aims to postpone the need for prosthesis and potential prosthesis revisions. Long-term, it is essential to continue monitoring the stability of bone structure and observe radiological evidence of femoral bone tissue regeneration to assess the ongoing effectiveness of stem cell therapy and whether there will be a need for a new approach.

Significant research by Daltro et al. (2008) illustrates how such monitoring can unveil more promising outcomes, as exemplified in the case described here. Over a span of 4 years, notable regeneration in the femoral head is evident, transitioning from FICAT IV to FICAT III classification. In cases of osteonecrosis of the femoral head secondary to sickle cell anemia, even the need for surgery remains uncertain. Neumayr et al. (2006) compared isolated physiotherapy with physiotherapy combined with simple decompression and found no significant differences in disease progression. However, in the literature, it is possible that patients with early-stage osteonecrosis of the femoral head (up to Ficat II) may have better pain relief, functionality, and quality of life with decompression with stem cell grafting (Saini et al., 2023; Wang et al., 2024).

In pediatric patients, Baghdadi et al. (2023) evaluated the use of bone marrow mesenchymal stem cell concentrate implantation in 23 patients with a mean age of 15.8 years. There was an improvement in the Harris Hip Score of these children and a decrease in pain to almost zero after 3 months of surgery. At the end of the follow-up, this pain score increased but did not return to baseline. Only three patients required hip arthroplasty subsequently.

It is crucial to note that patients with sickle cell anemia generally have a reduced life expectancy compared to the general population, making hip function improvement and pain control especially important, as many are in their economically active years (Nicholson et al., 2011; Tonino et al., 2013; Lopes et al., 2022). In children, this challenge is even greater, as the impact of early prosthetic placement or living with pain directly interferes with quality of life. Thus, cellular therapy would be of great value for treating these patients, delaying the need for prosthesis and improving pain scores (Daltro et al., 2015).

The clinical implications of these classifications cannot be underestimated. The high operability rate has the potential to directly impact clinical decision-making, with implications for patients’ quality of life. However, the limitations of the classifications, such as subjectivity in image interpretation and interobserver variability, must be carefully considered. The discussion should extend beyond the current findings, exploring how these classifications can be enhanced to improve diagnostic accuracy and patient care, as well as their impact on public health in terms of medical resources and costs.

The use of stem cells in research is widely encouraged, provided it complies with established ethical and regulatory guidelines, including approval by research ethics committees. Stem cell research is considered fundamental for advancing scientific knowledge and developing new therapies. Patients and healthcare professionals should exercise caution and seek treatments that comply with current regulations, ensuring that any clinical application of stem cells is carried out ethically and based on robust scientific evidence.

### 4.1 Study limitations

The study presents some limitations that should be considered when interpreting the results. The sample was restricted to patients from a single healthcare institution in Brazil, which may limit the generalizability of the findings to other populations. Additionally, the study design as a non-randomized clinical trial and the relatively small sample size may compromise the external validity and the ability to detect significant differences in clinical outcomes. The lack of a control group and limited patient follow-up also pose challenges in interpreting the results and assessing the long-term safety and efficacy of cell therapy. While the results suggest associations between age and treatment outcomes, there is a need to carefully consider other potential variables and inherent methodological limitations of a non-randomized study. It's possible that pediatric patients with femoral head collapse may not benefit as much from this approach.

## 5 Conclusion

The findings of this study underscore the potential of stem cell therapy as a promising intervention for early-stage osteonecrosis, particularly in younger patients. Notable benefits, such as significant pain alleviation, disease stabilization, and even regression of osteonecrosis, were observed, with pediatric cases demonstrating enhanced bone recovery. These results highlight the significant therapeutic potential of stem cell therapy in improving patient outcomes and quality of life.

This study represents a significant contribution to the medical field, providing a solid foundation for future research endeavors aimed at refining and standardizing stem cell therapy for femoral head osteonecrosis in patients with sickle cell anemia. Given the substantial risks associated with prosthetic use in pediatric cases, the importance of exploring alternative treatments like stem cell therapy cannot be overstated. Furthermore, the establishment of clinical trials comparing stem cell therapy with other conventional treatments will be crucial in further elucidating its efficacy and establishing it as a standard of care. This technological advancement instills hope for an improved quality of life for individuals grappling with this challenging condition, highlighting the critical need for ongoing research and interdisciplinary collaboration to propel medical advancements.

## Data Availability

The original contributions presented in the study are included in the article/Supplementary material, further inquiries can be directed to the corresponding author.
